# Adapting clinical practice guidelines for diabetic retinopathy in Kenya: process and outputs

**DOI:** 10.1186/s13012-018-0773-2

**Published:** 2018-06-15

**Authors:** Nyawira Mwangi, Muchai Gachago, Michael Gichangi, Stephen Gichuhi, Kibata Githeko, Atieno Jalango, Jefitha Karimurio, Joseph Kibachio, Lawrence Muthami, Nancy Ngugi, Carmichael Nduri, Patrick Nyaga, Joseph Nyamori, Alain Nazaire Mbongo Zindamoyen, Covadonga Bascaran, Allen Foster

**Affiliations:** 1 Kenya Medical Training College, Nairobi, Kenya; 2 Department of Ophthalmology, University of Nairobi, Nairobi, Kenya; 3 Ophthalmic Services Unit, Ministry of Health, Nairobi, Kenya; 4 Upper Hill Eye and Laser Centre, Nairobi, Kenya; 5 Kabarak University, Nakuru, Kenya; 6 Division of Non-Communicable Diseases, Ministry of Health, Nairobi, Kenya; 7 Kenya Medical Research Institute, Nairobi, Kenya; 8 Kenyatta National Hospital, Nairobi, Kenya; 9 The Fred Hollows Foundation, Nairobi, Kenya; 10 PCEA Kikuyu Eye Hospital, Kikuyu, Kenya; 11 London School of Hygiene and Tropical Medicine, London, UK

**Keywords:** Clinical practice guidelines, Diabetes mellitus, Diabetic retinopathy, Guideline development, Guideline adaptation, Kenya

## Abstract

**Background:**

The use of clinical practice guidelines envisages augmenting quality and best practice in clinical outcomes. Generic guidelines that are not adapted for local use often fail to produce these outcomes. Adaptation is a systematic and rigorous process that should maintain the quality and validity of the guideline, while making it more usable by the targeted users. Diverse skills are required for the task of adaptation. Although adapting a guideline is not a guarantee that it will be implemented, adaptation may improve acceptance and adherence to its recommendations.

**Methods:**

We describe the process used to adapt clinical guidelines for diabetic retinopathy in Kenya, using validated tools and manuals. A technical working group consisting of volunteers provided leadership.

**Results:**

The process was intensive and required more time than anticipated. Flexibility in the process and concurrent health system activities contributed to the success of the adaptation. The outputs from the adaptation include the guidelines in different formats, point of care instruments, as well as tools for training, monitoring, quality assurance and patient education.

**Conclusion:**

Guideline adaptation is applicable and feasible at the national level in Kenya. However, it is labor- and time -intensive. It presents a valuable opportunity to develop several additional outputs that are useful at the point of care.

**Electronic supplementary material:**

The online version of this article (10.1186/s13012-018-0773-2) contains supplementary material, which is available to authorized users.

## Background

The first definition of clinical practice guidelines (CPG), hereafter referred to as “guidelines,” was provided by the Institute of Medicine (IOM) in the USA in 1990: “systematically developed statements to assist practitioners and patient decisions about appropriate healthcare for specific circumstances” [1]. Guidelines-related initiatives have subsequently increased globally since the 1990s. This definition was revised in 2011 to: “statements that include recommendations to optimize patient care that are informed by a systematic review of the evidence and an assessment of the benefits and harms of alternative care options” [2]. Guidelines constitute one tool for good decision-making in clinical practice, which has potential to reduce variations in health care and its cost. Although a plethora of barriers may compromise their effectiveness, guidelines are instruments to improve the quality of care.

Guideline adaptation is potentially an efficient alternative to de novo guideline development, particularly in resource-constrained contexts [3]. Adapting guidelines to suit a local context may also improve local uptake of the guidelines [4, 5]. Adaptation requires an active, systematic, and participatory process [4] that preserves the integrity of the transferable evidence-based recommendations. Although this adaptation process is context-specific and may not be transferable or generalizable, it needs to be systematic, explicit, transparent, rigorous, and reproducible. The ADAPTE and Practice Guideline Evaluation and Adaptation Cycle (PGEAC) framework of adaptation are validated approaches to conduct and document this process [4].

The Institute of Medicine [2] has described eight attributes of good guideline development. These are (a) validity, (b) reliability and reproducibility, (c) clinical applicability, (d) clinical flexibility, (e) clarity, (f) documentation, (g) development by a multidisciplinary process, and (h) plans for review. Guidelines are likely to reflect these attributes when they are developed via a transparent process by a multidisciplinary team without potential bias and conflicts of interest, and supported by a systematic review of the evidence [2].

This paper describes the process involved in adapting the diabetic retinopathy (DR) guidelines for Kenya, in order to assist others undertaking a similar endeavor.

The STEPwise survey [6] for risk factors of non-communicable diseases in 2015 reported that diabetes mellitus (DM) affects an estimated 2% of the Kenyan population aged 18–69 years, with the highest proportion (5%) being in the 45–59 years age group. Every person living with diabetes (PLWD) is at risk of potentially blinding diabetic retinopathy (DR). In turn, visual loss from DR is associated with additional morbidity, such as falls, fractures, and difficulties with taking medications. Both DM and DR are associated with significant morbidity, mortality, and excess health care costs. The prevalence of DM is predicted to rise steeply over the next decade [7], and consequently DM and DR are important public health concerns.

Effective and quality service delivery in relation to DR in Kenya is required within the existing health system [8–10]. Currently, there are notable gaps in DR screening, diagnosis, referral, treatment, and follow-up. Although screening and laser treatment are cost-effective interventions for prevention of blindness from DR [11], there are inequities in access to them. Some of the services are underutilized for a variety of reasons, while some of the services delivered are of insufficient quality. This disparity is linked to multiple supply and demand factors, such as variation in referral practices of diabetes care providers, screening practices of eye care providers, integration of services, and level of awareness of patients [9, 12, 13].

Clinical guidelines offer recommendations to improve service delivery, advocate for resources, leverage existing resources, and improve outcomes. Implementing evidence-based practice guidelines for DR is thus vital to address the gaps and prevent blindness from DR. International guidelines for this purpose exist, but there are no published local guidelines. This guideline adaptation aimed to address this lack of national guidelines. We envisaged providing a user-friendly guideline that describes appropriate care based on the best available scientific evidence.

## Methods

We relied on adaptation instead of de novo development of the guideline in order to avoid duplication of effort, to use the available resources cost-effectively, and to facilitate customization of the guidelines prepared for other income and health system settings to reflect local context.

The process of standardizing clinical practice recommendations for DR in Kenya began over a decade ago. Several guideline documents have been produced although none has been formally published as a national guideline. Our reflection was that opportunity costs, turnover of experts involved in the process, and other contextual factors might have slowed down further development of the guidelines. The methodology discussed here is that followed over the last 2 years leading to the production of the published guideline. However, we expect that a similar process was undertaken in the previous period.

**Fig. 1:**
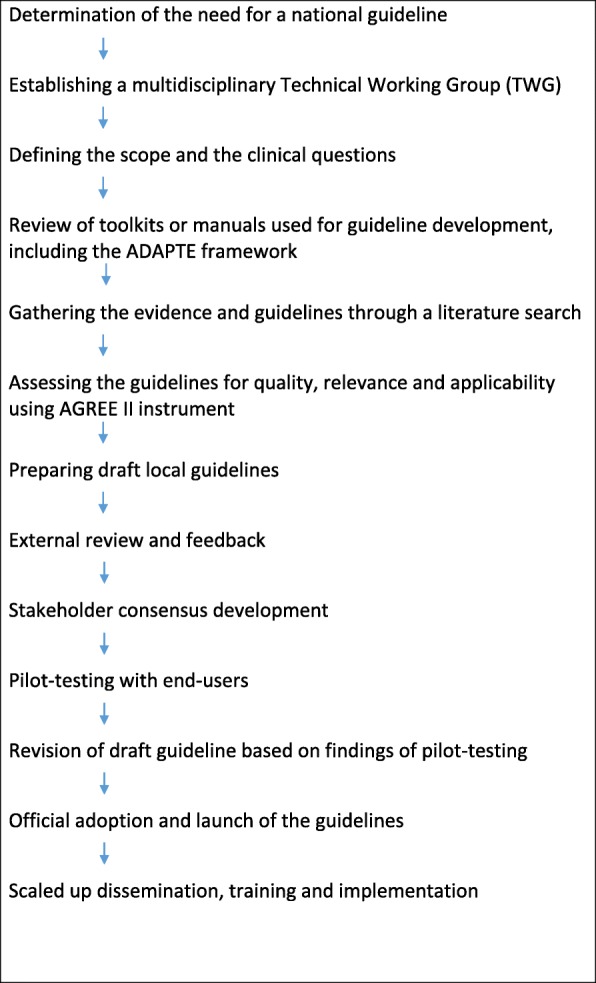
Schema for the methods used in guideline adaptation

The adaptation process has been systematic, consultative, and guided by a technical working group (TWG). Several widely used toolkits and guidelines provided a point of entry [14–20]. We followed the tasks of adaptation, as applied within the ADAPTE framework, although some of the tasks were synchronized and we often had to return to previously completed steps. The ADAPTE process is a well-known framework for guideline adaptation, which consists of 3 phases and 24 steps. Seven core principles underpin this framework, and the TWG adopted them for this adaptation [14]. Figure 1 provides a simplified schema of our methods.

The identification of the need for the DR guidelines by stakeholders prompted the Ophthalmic Services Unit to constitute a steering team of five members. This team prepared terms of reference and a list of potential members for the TWG. At the first few meetings of the TWG, we discussed the following: the need for the guidelines, the feasibility of guideline development, the required expertise, funding, work plan, outputs, and role definition of the members. In subsequent meetings, the topic and clinical questions were defined.

We identified the methodological resources, the clinical guidelines, and the evidence for effectiveness of various DR interventions through a literature search on various databases including Cochrane Library, ELDIS, Embase, Global Health, and PubMed. We also searched the websites of agencies that develop these resources. The search strategy (Additional file 1) was limited to reports published in English from 2000 up to date.

Two TWG members conducted the literature search and recorded the characteristics and content of retrieved guidelines. Guidelines that did not meet the predefined inclusion and exclusion criteria were eliminated. Two reviewers assessed the quality of the retrieved guidelines using the AGREE II instrument and presented the findings in a TWG meeting. All TWG members participated in the assessment for currency, content, consistency, acceptability, and applicability of the recommendations. Following consensus on the results of the assessment, guidelines suitable for adaptation were selected.

The clinical guidelines that were selected for reference were the American Diabetes Association (ADA) Standards for Medical Care in Diabetes [21], International Council of Ophthalmology (ICO) guidelines for diabetic eye care [22], the Royal College of Ophthalmologists’ diabetic retinopathy guidelines [23], and the Canadian Diabetes Association’s retinopathy guidelines [24]. We chose the ADA and ICO guidelines as the prototypes for DM and DR guidelines respectively and collated evidence from Cochrane systematic reviews relevant to diabetic retinopathy. None of the guidelines contained an adaptation template for different contexts.

We utilized the AGREE II (Appraisal of Guidelines Research and Evaluation II) instrument to assess the quality of the clinical guidelines. This instrument consists of 23 items grouped into six domains: (a) scope and purpose, (b) stakeholder involvement, (c) rigor of development, (d) clarity and presentation, (e) applicability, and (f) editorial independence.

The following additional guidelines were also reviewed, so as to identify any potential conflict in recommendations for care of diabetes and other comorbidities: Kenya national guidelines for management of diabetes [25], Kenya national strategy for the prevention and control of non-communicable diseases [26], and International Diabetes Federation’s diabetes eye health guide for health professionals [27]. Similarly, we reviewed previous drafts of local DR guidelines.

Draft guidelines were prepared by the TWG and circulated via email to all members for review. Three drafts were circulated, with the final draft also being circulated to external reviewers to assess content validity, clarity, and applicability. The TWG evaluated the final draft guidelines for the quality requirements of the AGREE II instrument prior to release. A consensus stakeholders meeting approved the final draft.

Pilot testing was conducted in different health care settings in purposively selected counties that differed in characteristics that may influence applicability. We collected feedback on the usability through interviews, reports, and observation. Dissemination of guidelines was done through county coordinators, conferences, training institutions, professional associations, social media, and distribution of print material.

## Results

Table 1 shows the results of the adaptation process in each of the steps of the ADAPTE framework. We applied the guiding principles as exhibited in Table 2. We further applied the AGREE II instrument to ensure quality of our draft guideline as reported below.

**Table 1:** Adapting the guidelines using the ADAPTE process

Step	Activity	Result
Phase I Set up		
1.	Establish a resource team	The Ophthalmic Services Unit constituted a steering team of five members which developed the terms of reference and prepared a list of 25 potential members for the technical working group (TWG), who were subsequently invited to the group
2.	Determine criteria for selection and select a topic using criteria	DR was selected because it is a public health concern and there is variation in standards of care
3.	Check if adaptation is feasible	Evidence-based guidelines were already in use internationally, and there was high interest from the Ministry of Health, clinicians, and other users to develop guidelines
4.	Identify necessary resources and skills	There was high level of commitment by members of the TWG. The Fred Hollows Foundation committed to provide funds, and the required expertise was available: retinal specialists, public eye health specialists, endocrinologists, diabetes educators, epidemiologists, search and retrieval of information, critical appraisal, research, policy, guideline development, and eye health systems. A need for input from other professions in the multidisciplinary care team for type 1, type 2, and gestational diabetes was identified
5.	Complete tasks of the set-up phase	Members of the group decided to function as a working group coordinated by the Ophthalmic Services Unit. A set of guiding principles to foster development of the guidelines was adopted (Table 2). Potential conflicts of interest were explored, and there were none to declare
6.	Write the plan for adaptation	A timeline for completion, list of additional resource persons to be included, list of outputs to be developed in conjunction with the guidelines and task allocation among the TWG members were agreed upon
Phase II Adaptation		
7.	Determine and clarify the question	A PIPOH summary was prepared (Table 3). The areas of interest for standards of care were determined as screening, diagnosis and management of DR, and the management of DM in relation to DR, within the existing care pathway for PLWD in the Kenyan health system
8.	Search for guidelines and other relevant documentation	The TWG searched for relevant DR guidelines and evidence on DR interventions in systematic reviews
9.	Screen the retrieved guidelines and record their characteristics and content	The recommendations of the guidelines for screening, diagnosis and management of DR, and the management of DM in relation to DR was reviewed, extracted, and compiled in summary tables. Evidence from Cochrane systematic reviews was also reviewed
10.	Eliminate a large number of the retrieved guidelines using the AGREE instrument	The rigor dimension of the AGREE II tool was utilized to eliminate guidelines that did not meet the stipulated criteria
11.	Assess the quality of the guideline	The AGREE II instrument was used to scrutinize the quality of the guidelines
12.	Assess the currency of the guideline	The guidelines retrieved were sufficiently current, and we did not identify any new evidence
13.	Assess the content of the guideline	Recommendations for screening, diagnosis, grading, referral and treatment were examined and did not differ significantly between guidelines
14.	Assess the consistency of the guideline	There was clear consistency between the evidence from systematic reviews, the interpretation of the evidence, and the recommendations in the guidelines in all the areas of interest
15.	Assess the acceptability and applicability of the recommendations	Care was taken to ensure the recommendations are not in conflict with other local guidelines and to appraise the implications of the guidelines on health service delivery
16.	Review assessments	The results of the assessment of the guidelines were discussed in meetings of the TWG
17.	Select among guidelines and recommendations to create an adapted guideline	The ICO guideline for DR was the main guideline used because the recommendations compared well with the other high-quality DR guidelines and the practice-based recommendations were well-stated
18.	Prepare a draft of the adapted guideline	The facilitators of the working group compiled the results of the deliberations and wrote the draft guideline document
Phase III Finalization		
19.	Seek feedback on the draft guideline from those who would be using it	Three revisions of the draft were circulated for comment to TWG members as well as surgeons, pediatricians, ophthalmologists, Kenya Defeat Diabetes Association, vitreoretinal surgeons, physicians, diabetes educators for agreement and identification of gaps
20.	Consult with endorsement bodies	The Ministry of Health adopted the guidelines
21.	Consult with developers of guidelines used as sources	No substantive changes were made to recommendations so this step was not undertaken
22.	Acknowledge source documents	The key guideline documents and other resources used have been acknowledged through attribution
23.	Plan for aftercare of the adapted guideline	A review date was planned for 5 years. Monitoring indicators were also identified. Pilot-testing has been used to check for usability. Distribution will be through electronic and print copies
24.	Produce a final document of the guideline and other outputs	The following additional outputs were produced (along with the guideline): posters and brochures for patient information, posters, brochures and checklist to be used by clinicians, workshop slides for training health workers, quality assurance guidelines

**Table 2:** Guiding principles for guideline adaptation

	Guiding principle	Indicator
1.	Respect for evidence-based principles in the development of guidelines	The evidence on which the recommendations are based is included in the guidelines
2.	Ensuring that the quality of guidelines is high	Well-known frameworks for guideline development were used to guide and assess the quality of the adaptation process
3.	Participation of key stakeholders to foster acceptance and ownership of the adapted guideline and ultimately promote its use	The involvement of stakeholders was acknowledged in reports of the adaptation process
4.	Consideration of context during adaptation to ensure relevance for local practice and policy	The context of application of the guidelines has been explicitly stated and the content adapted for the Kenyan health system
5.	Transparency to promote confidence in the guideline development process	The methodology in the adaptation process has been documented so that it is accessible and reproducible
6.	Flexibility to accommodate specific needs and circumstances in the health system	The guideline presents recommendations for diverse categories of PLWD (such as those with different stages of DR or comorbidities) who receive service in different clinical settings
7.	Respect for and acknowledgement of guideline materials used as sources	Citation and referencing have been used to acknowledge all source documents

### Scope and purpose

The TWG’s first task was to define the scope. The main options were to include only DR or diabetic eye disease as a whole. The consensus was to limit the scope to DR because of its unique natural history and public health implications. The overall objective of the guideline is to reduce the proportion of PLWD who go blind due to diabetic retinopathy in Kenya through interventions for prevention, early detection, and effective treatment of DR. The adaptation process aimed to reduce inappropriate variation in screening and treatment, to provide a rational guide for referral, and to use the diabetes care and eye care resources efficiently to meet these goals. The recommendations needed to be germane to the social context, the patient pathway, and the referral systems in addition to being capable of integration into the routine workflow. The Population, Intervention, Professions, Outcomes and Health care system (PIPOH) summary (Table 3) defined the clinical questions addressed in the guidelines.

**Table 3:** PIPOH summary of the clinical questions

	Parameter	Specification
P	Population	All patients with diabetes mellitus who are aged ≥ 12 years
I	Intervention	Screening, diagnosis, referral, and management of diabetic retinopathy
P	Professions (target users)	Primary care workers, diabetes care providers, eye care workers, administrators, policy-makers
O	Outcomes	All persons living with diabetes are screened for DR at least annually and blindness from DR is prevented
H	Health care setting	Community, Primary, Secondary, and Tertiary level health care settings

### Stakeholder involvement

The Ophthalmic Services Unit at the Ministry of Health convened a steering group of five members. They drafted the terms of reference for a task-oriented TWG, which were to (1) determine the scope and focus of the required guidelines, (2) appraise the evidence and recommendations in existing DR guidelines, (3) develop the national guideline, and (4) craft messages to be used at the point of care, to influence practice.

The steering group identified 25 potential members of the TWG, based on the criteria of diverse expertise, experience, representation of multiple stakeholder groups, and commitment to the process, all aimed at increasing both internal and external validity of the guidelines. These members received personal invitations to participate. An average of 15 were active members of the TWG at any given time, but the others remained involved on the periphery and received frequent reminders to participate remotely. Participation was through attending meetings, email and telephone correspondence, face-to-face consultations, availing resource documents, reviewing drafts, providing evidence, and informal consultations. This proactive integrative and flexible approach was designed to ensure ownership, external validity, and the involvement of end users of the guideline.

The TWG members were all volunteers with other clinical, educational, administrative, and policy roles related to DM and DR in public, private, or faith-based health facilities, academia, ministry of health, and professional organizations. Participation on a volunteer basis inferred limitation of availability, though additionally, it implied indirect institutional participation of the employer. They had diverse expertise including clinical, public health, research, epidemiology, literature search, systematic reviews, and health systems. Differences in opinion were encountered in the deliberation of some recommendations, particularly regarding the role of different cadres in making DR treatment decisions and delivering treatment. This was resolved through varied strategies: expressing judgements about values and risks, making reference to regulation, reviewing the evidence for role specification, and adapting the role definition prescribed by the source guidelines and informal consensus techniques.

We did not employ a research assistant, because the team had skills in literature review, recent systematic reviews on interventions for DR were available and the existing guidelines were current. The team did not have a health economist and did not conduct an economic appraisal. As the guidelines were in English, we did not require expertise in foreign languages.

The TWG considered it is important to include patients’ values and perspectives in the guidelines. A patient group was invited and PLWD who are clinicians were included, but despite our efforts, we did not succeed in having patients directly participate in the adaptation. We also aspired to have the participation of large groups of PLWD in a way that adequately represents the diversity of perspectives of PLWD from different geographical locations, social strata, and stages of disease. Since we do not have a comprehensive database of PLWD in the country, this was not feasible.

### Rigor of development

We obtained high quality and current international guidelines. We examined the methods and the quality of the evidence used to formulate the recommendations for interventions for DR. We also considered the implications for resources and health service delivery in Kenya. Further, we searched for any recent evidence from systematic reviews and for relevant domestic research. In the absence of this, and judging the recommendations current and evidence-based, we incorporated them in our guidelines. The draft guidelines were subsequently reviewed by external multidisciplinary reviewers and pilot-tested in various health facilities. The guideline will be updated in 5 years to incorporate any new evidence that will have emerged.

### Clarity and presentation of the guideline

We used the Conference for Guideline Standardization (COGS) checklist [20] as a guide to the content that needed documentation, although we excluded those items on the list that we did not consider necessary. The adapted guideline also includes additional information that was not in the international guidelines, such as the pattern of diabetes in Kenya, integrating DM and DR services, dissemination, and review plans.

In writing the guidelines, we avoided vague, nonspecific, or ambiguous terms and phrases. We aimed to produce a user-friendly guideline in which the precise recommendations are easily identifiable and clear, and the formatting is appropriate.

### Applicability

We recognized the facilitators and barriers to the application of this guideline in the Kenyan health system. To overcome the barriers, the guideline provides tools to facilitate its implementation at the point of use. These include workshop slides for training guideline implementers. Flexible 1- day training programs have been executed at implementing health facilities, conferences and training institutions, in conjunction with guidelines dissemination. The potential resource implications (equipment, staff, and training) and resultant work burden of applying the recommendations were considered. A monitoring and evaluation plan has also been included to assess adherence to recommendations and the outcomes of the implementation.

We required data on the costs of DR services in Kenya, but we did not undertake this as the Division of Non-Communicable Diseases had recently undertaken costing for diabetes services, including DR services. We lacked a costing model for guideline adaptation at the start of the exercise, but in our experience, the largest cost of the adaptation process was the production, pilot-testing, dissemination, and implementation of the print outputs. This may be reduced with progressive enhancement of digital literacy of the users and increased utilization of the electronic resources.

### Editorial independence

The basic logistic needs of the adaptation process (administrative and meeting costs), as well as the implementation costs, were funded by The Fred Hollows Foundation. The funders did not influence the content of the guideline. There was a 100% consensus on the desired outcome of the guidelines, which is prevention of blindness from DR. Members and funders did not have any conflict of interest with respect to this outcome.

#### Context-specific modifications

Unlike the reference guidelines, the Kenyan guideline is designed for use by both diabetes and eye care clinicians, as well as other stakeholders in eye care. The population of interest is all PLWD aged 12 years and over, without any exceptions. The guideline attempts to take care of various complexities of service delivery. The role of different cadres in the screening, diagnosis, and treatment of DR is highlighted. Noting the variability in access to required equipment and skills, a referral mechanism has been determined through mapping of the services available in different facilities in the country. We found it practical to constantly relate the guideline to the patient pathway. Additional specifications have been made on linking diabetes care and eye care services, clinical governance for the services, and using the health information management systems (HIMS) to monitor the effect of implementation of the guidelines.

#### Outputs

This process has led to several outputs: (i) the national guidelines in various formats—print copies, electronic version, and an executive summary of the recommendations. The guideline has a national coverage and applies to persons with any type of diabetes.' (ii) quality assurance guidelines; (iii) mapping of DR services in the country; (iv) posters and leaflets for patients; (v) posters and checklists for clinicians; (vi) workshop slides for training health workers; and (vii) monitoring and evaluation tool. These outputs are to be used at the point of care by diabetes and eye care clinicians, as well as by administrators and policy makers. They were chosen because they were perceived to increase convenience of users and to intensify user adoption of the guidelines. They are in English, but they can be translated. An executive summary of the guidelines is published in a separate paper [12].

Feedback from pilot-testing indicated that the guideline is useful in various clinical and geographic settings in the country. It served an educational role for clinicians and reduced missed opportunities for screening and referral. The demand for the print outputs continued after pilot-testing. The point-of-use outputs were reported to boost user satisfaction because they contained simplified key messages for different users. During prospective collection of feedback, the lack of a tool to guide integration of diabetes and eye care services has been identified as a gap, and its development is being considered.

## Discussion

The process of DR guideline development in Kenya has taken several iterative episodes. This trajectory may reflect the intensive work that guideline adaptation entails, as well as the capacity building that has resulted over that process. This experience is not unique to this initiative; long timelines have been reported in other guideline initiatives in the same context [28]. Contextual factors such as transitions in the guideline development team or critical leadership may result in delays, repetition of effort, and modification of approach.

We did not experience a shorter time scale for adaptation compared to the 2–3 year period suggested for de novo development or shorter timelines for adaptation [29]. This could be because we did not conduct this guideline development process continuously and the team of experts had other primary engagements. However, it is evident that the adaptation approach also requires a heavy time commitment. From our experience, which concurs with the literature, it is an essential prerequisite to realistically determine the workload, resources, access to expertise, and the need for dedicated leadership [29].

The diverse expert skills and commitment of our multidisciplinary TWG are a recognizable success factor for our initiative. This is pertinent for both the internal and external validity (generalizability) of the guidelines [4, 17]. As adaptation requires significant investment from this team, the selection of potential TWG members is a pivotal priority step. Kenya being one of the countries with facing health workforce crisis [30], the major drawback we faced was availability of the TWG members to attend face to face meetings, as they had competing clinical and managerial responsibilities. This was predictable and inexorable, necessitating strategies to ensure group functioning was not interrupted.

The integrative participation method, which allowed both in-person participation and remote participation of the working group, helped to mitigate this constraint. This may have provided impetus to the process and achievement of the outcome. The enabling factor was that both electronic communication and face-to-face meetings were feasible, allowing for flexible engagement.

Involving patients in the process of guideline development is recommended [1, 29], because their opinions about the process of investigation and treatment and their outcomes are often quite contrary to the views of professionals. The lack of practical methods for engagement of PLWD precluded it. Although there are resources to guide this, such as the toolkit from Guideline International Network [16], local literature or precedence to guide such a process is lacking. Despite invitation of patient representatives to attend meetings and to review the drafts, we did not get this direct input. This may be because this type of involvement is not instinctively consistent with patient expectations or felt needs in our setup. Recruiting diverse groups of PLWD in a representative manner in a setting without comprehensive databases also requires contextual strategies. These limitations call for further local research.

We found that the guideline adaptation had the pattern of back and forth interlinked steps. We did not follow the ADAPTE steps sequentially as a stringently linear progressive and prescriptive tool. Further, even with the use of methodological tools, we found that there is need to maintain focus so that the process is not staggered. Clarity of the scope and significance to the patient pathway helped to maintain focus and continuity. Focus helped to avoid attrition, considering that universal completion of guideline development is not the norm [1].

Similar to de novo development, adaptation requires a review of the evidence and explicit use of valid evidence [15]. It would have enriched the process if we had additional domestic evidence. There is need for local research to fill gaps in scientific knowledge regarding interventions for DR in this population and health system. Local evidence on economic analysis such as cost-minimization and cost-utility evidence of the interventions is also necessary.

Adaptation itself has cost implications, and although we did not have a large dedicated budget for the process, we found it important to have a budget for the variable costs. In the absence of funding for fixed costs, we cannot provide an estimate of the funding required. Such estimates would help to calculate the cost-effectiveness of the process, considering the opportunity costs, and comparison with the cost of de novo development. The interventions described in the guidelines are clinically effective, but we need to investigate whether these interventions and the process of guideline development are also cost-effective in our setting. At present, we assume that guidelines augment the efficiency of DR services and optimize value for every shilling invested in the health system. In order to balance cost and accessibility of services, the guidelines promote the use of existing resources while aspiring to progressively mobilize the range of resources that are recommended.

National guidelines for various conditions may contain conflicting recommendations, which can be confusing for clinicians. This is especially the case for PLWD, as they often have comorbidities. We avoided such discrepancies by reviewing the other diabetes-related guidelines, and we recommend this as an important step in adaptation.

The output from the adaptation process is a guideline that is different from the generic guidelines (or source guidelines) and contains additional information that will be useful for the target user. An additional benefit is the production of additional tools for use at the point of care. This shows that the role of guideline adaptation is not limited to endorsing generic recommendations.

This initiative has coincided with other DR activities, such as the initial steps towards implementing regular retinal screening for PLWD attending diabetes services. This may have contributed to its success, and we can further leverage on this to market the guidelines. Given that countries in the African region, particularly the Eastern, Central, and Sothern African region may face similar needs to develop guidelines, a network or collaboration of sharing and learning may be an efficient approach to develop them.

## Conclusions

Guideline adaptation is a structured investment -intensive process that is feasible at the country level. Rigor and focus are important in this process. The ADAPTE process and AGREE II instrument are valuable tools for this process, though it would be helpful for the generic guidelines to have an adaptation template for other contexts.

Multiple informational, technological, economic, social, and professional variables influence the effectiveness of guideline adaptation. Beyond the utility of this process in producing the outputs we required, it could also be useful to inform the development of other guidelines in similar contexts. Our experience has helped to provide insights on the use of the adaptation methodology in the African context. We have also identified guideline development as a potential area for collaboration.

Involvement of the end user of the guidelines (diabetes and eye health clinicians) in this adaptation process aims to increase adherence to the guidelines. We expect that DR services that were not routinely available to PLWD in Kenya will now become accessible as a response to the guidelines.

### Implications for practice

Availability of a national guideline is a necessary but not sufficient impetus to standardize patient care. The extent to which the prevention of blindness from DR is realized will depend on the effectiveness of guideline dissemination and implementation, in tandem with other interventions.

### Future research

An economic analysis is required to determine whether guideline adaptation is cost-effective. Research evidence is also required to determine the effective methods of involving patients, such as DR patients, in the adaptation process. In addition, the effectiveness of the guidelines in reducing DR blindness will need evaluation.

## Additional file

Additional file 1: Search strategy for “Adapting Clinical Practice Guidelines for Diabetic Retinopathy in Kenya” (PDF 568 kb)

## Abbreviations

ADA: American Diabetes Association
AGREE: Appraisal of Guidelines for Research and Evaluation
COGS: Conference for Guideline Standardization
CPG: Clinical practice guideline
DM: Diabetes mellitus
DR: Diabetic retinopathy
ICO: International Council of Ophthalmology
IOM: Institute of Medicine
PGEAC: Practice Guidelines Evaluation and Adaptation Cycle
PIPOH: Population, Intervention, Profession, Outcomes, Health care system
PLWD: People living with diabetes
TWG: Technical Working Group
