# Supplementary material for: Adapting clinical practice guidelines for diabetic retinopathy in Kenya: process and outputs
Source: Implement Sci. 2018 Jun 15;13:81. doi: 10.1186/s13012-018-0773-2 (PMC6003001; doi:10.1186/s13012-018-0773-2)
Supplement: Supplementary file 1 — Search strategy for “Adapting Clinical Practice Guidelines for Diabetic Retinopathy in Kenya” (PDF 568 kb) [file 13012_2018_773_MOESM1_ESM.pdf]

Additional file 1

Adapting Clinical Practice Guidelines for Diabetic Retinopathy in Kenya: the literature search strategy

Nyawira Mwangi[1,11]✉, Muchai Gachago[2,4], Michael Gichangi[3], Stephen Gichuhi[2], Kibata Githeko[4], Atieno Jalango[5], Jefitha Karimurio[2], Joseph Kibachio[6], Lawrence Muthami[7], Nancy Ngugi[8], Carmichael Nduri[9], Patrick Nyaga[8], Joseph Nyamori[2], Alain Nazaire Mbongo Zindamoyen[10], Covadonga Bascaran[11], Allen Foster[11]

[1]Kenya Medical Training College, Nairobi, Kenya

[2]Department of Ophthalmology, University of Nairobi, Nairobi, Kenya

[3]Ophthalmic Services Unit, Ministry of Health, Nairobi, Kenya

[4]Upper Hill Eye and Laser Centre, Nairobi, Kenya

[5]Kabarak University, Nakuru, Kenya

[6]Division of Non-Communicable Diseases, Ministry of Health, Nairobi, Kenya

[7]Kenya Medical Research Institute, Nairobi, Kenya

[8]Kenyatta National Hospital, Nairobi, Kenya

[9] The Fred Hollows Foundation, Nairobi, Kenya

[10]PCEA Kikuyu Eye Hospital, Kikuyu, Kenya

[11]London School of Hygiene and Tropical Medicine, London, United Kingdom

Topic and Scope

Clinical guidelines for screening and management of diabetic retinopathy

Key concepts

1. Clinical practice guidelines
2. Diabetic retinopathy
3. Screening
4. Management

Databases searched

1. Cochrane Library
2. ELDIS
3. EMBASE
4. Global Health
5. PubMed
6. Grey Literature
    a. Guideline development groups: National Institute for Clinical Excellence (NICE), Agency for Health Care Research and Quality (AHRQ), Scottish Intercollegiate Guidelines Network (SIGN
    b. Websites: AGREE, GRADE, World Health Organization, Ministry of Health Kenya, Relevant professional associations (such as American Diabetes Association, International Diabetes Federation, Canadian Diabetes Association, International Council of Ophthalmology and Royal Colleges of Ophthalmologists)
    c. Guidelines International Network Database

Search process

1. Each database was searched individually, and the search strategy was adapted to suit each database.
2. A title and abstract search as well as subject heading search was conducted for each concept. This was followed by a search using a combination of both.
3. References cited within key literature were also hand-searched.
4. Google and Google scholar internet search engines were also used in the search using terms that included 'diabetic retinopathy' and 'clinical guidelines'.
5. Technical Working Group members also searched their personal libraries for any published or unpublished guidelines.

Inclusion criteria

1. Publication date of 2000 and later
2. Guidelines in English
3. Any geographical, resource or health care setting
4. Any category of the population living with diabetes
5. Any type of publication, such as book, book chapter, conference proceeding or journal article
6. Guideline content includes, but is not necessarily limited to recommendations on screening and management of diabetic retinopathy
7. Guideline articulates the evidence for each recommendation

Exclusion criteria

1. Guidelines that did not contain recommendations on screening and management
2. Guidelines that did not cite the evidence
3. Guidelines that focused on diabetes in general or on other complications of diabetes without giving particular attention to diabetic retinopathy
4. Guidelines not in English language

Search terms

Clinical guidelines	Diabetic retinopathy	Screening	Management
guideline*<br>clinical practice guideline*<br>CPG*<br>practice guideline*<br>consensus development<br>consensus statement*<br>clinical decision-making<br>clinical pathway<br>clinical protocol<br>evidence-based guidelines<br>practice pattern<br>practice parameter	diabetes adj5 (eye, eyes, ocular, blindness, visual impairment)<br>diabetic*<br>DR<br>diabetic eye disease<br>retinopathy<br>maculopathy<br>macula oedema<br>macula edema<br>sight loss<br>vision loss<br>blindness<br>blind*	examination<br>eye examination<br>ocular examination<br>retinal examination<br>clinic<br>risk factors<br>risk assessment<br>risk reduction<br>behaviour<br>ophthalmoscopy<br>fundoscopy<br>fundus photography<br>retinal imaging<br>diagnosis<br>classification<br>grading<br>grader*<br>evaluation<br>eye test*<br>vision test*<br>screening	care<br>treatment<br>referral<br>follow-up<br>prevention<br>prognosis<br>patient experience<br>management<br>monitoring<br>self-management<br>patient education<br>laser<br>photocoagulation<br>intravitreal injection*<br>vitrectomy
